# A novel resistance gene for bacterial blight in rice, *Xa43(t)* identified by GWAS, confirmed by QTL mapping using a bi-parental population

**DOI:** 10.1371/journal.pone.0211775

**Published:** 2019-02-12

**Authors:** Suk-Man Kim, Russell F. Reinke

**Affiliations:** 1 Strategic Innovation Platform, International Rice Research Institute, Los Baños, Philippines; 2 IRRI-Korea Office, National Institute of Crop Science, Rural Development Administration, Iseo-myeon, Wanju-gun, Jeollabuk-do, Republic of Korea; ICAR-Indian Institute of Rice Research, INDIA

## Abstract

Bacterial blight (BB) caused by the *Xanthomonas oryzae* pv. *oryzae* (*Xoo*) pathogen is a significant disease in most rice cultivation areas. The disease is estimated to cause annual rice production losses of 20–30 percent throughout rice-growing countries in Asia. The discovery and deployment of durable resistance genes for BB is an effective and sustainable means of mitigating production losses. In this study QTL analysis and fine mapping were performed using an F_2_ and a BC_2_F_2_ population derived from a cross with a new *R*-donor having broad spectrum resistance to Korean BB races. The QTL *qBB11* was identified by composite interval mapping and explained 31.25% of the phenotypic variation (*R*^*2*^) with LOD values of 43.44 harboring two SNP markers. The single major *R*-gene was designated *Xa43 (t)*. Through dissection of the target region we were able to narrow the region to within 27.83–27.95 Mbp, a physical interval of about 119-kb designated by the two flanking markers IBb27os11_14 and S_BB11.ssr_9. Of nine ORFs in the target region two ORFs revealed significantly different expression levels of the candidate genes. From these results we developed a marker specific to this *R*-gene, which will have utility for future BB resistance breeding and/or *R*-gene pyramiding using marker assisted selection. Further characterization of the *R*-gene would be helpful to enhance understanding of mechanisms of BB resistance in rice.

## Introduction

Rice bacterial blight (BB), caused by *Xanthomonas oryzae* pv. *oryzae* (*Xoo*), is one of major diseases causing severe production losses in most rice cultivation areas. The disease poses a continuous threat to rice production especially in South and Southeast Asia [1,2]. Developing resistant lines or cultivars is the most effective and economical way to control this disease, while minimizing use of chemical disease control and the attendant environmental concerns [3].

Damage from this major foliar disease has led to rice production losses of 20–30 percent [4]. Yield losses of up to 80–90% of the total yield in India and Philippines have been reported [5,6]. In general, the damage begins at the tillering stage and disease incidence increases with plant growth, with maximum incidence at the flowering stage [7]. The easiest way to prevent the disease is to apply chemical control. However chemical control of BB in the monsoon climate of Asia is impractical because it spreads rapidly, and once it occurs on a large scale effective control through chemical application is very difficult [8]. Additionally, it directly increases the cost of rice production and the widespread use of chemical carries environmental risks [9]. Therefore enhancing host resistance is considered the most effective strategy to achieve disease resistance in rice. To prevent breakdown of resistance by pathogenic variation in *Xoo*, as a result of the evolution of new pathotypes, pyramiding two or more effective resistance genes in developing rice cultivars is required for durable and sustainable BB resistance [10,11]. Moreover their characterization and availability of tightly linked markers will greatly facilitate effective breeding for resistance to BB.

To date more than 42 BB *R*-genes conferring resistance to *Xoo* have been identified in rice cultivars, wild relatives of rice, and mutation populations [12]. Of these, only nine genes (*Xa1*, *Xa3/Xa26*, *xa5*, *Xa10 xa13*, *Xa21*, *Xa23*, *xa25*, *and Xa27*) have been characterized at the molecular level and these encode various types of proteins suggesting multiple mechanisms of *R*-gene-mediated *Xoo* resistance [13–17]. Twelve R-genes (*Xa4*, *Xa7*, *Xa22*, *Xa30*, *Xa31*, *Xa33*, *xa34*, *Xa35*, *Xa39*, *Xa40*, *xa42 and Xa42*) have been fine-mapped based on morphological and molecular markers [12,18–20]. Of the complete spectrum of BB *R*-genes, 16 recessive genes (*xa5*, *xa8*, *xa9*, *xa13*, *xa15*, *xa19*, *xa20*, *xa24*, *xa25*, *xa26b*, *xa28*, *xa31*, *xa32*, *xa33*, *xa34*, and *xa42*) have been reported [7,20,21] and the remainder are dominant. The BB *R*-genes are distributed across 9 of the 12 rice chromosomes, with none reported on chromosomes 1, 9, and 10, while more than 8 BB *R*-genes were intensively clustered on chromosome 11. In general the major classes of *R-*genes are nucleotide-binding site-leucine-rich repeat (NBS-LRR) genes and the cell surface pattern recognition receptors [14,22]. Whereas most of the BB *R*-genes produce unique proteins and these products are not found in other plants except *Xa1* (NBS-LRR) and *Xa21*·*Xa26* (Receptor-like kinase).

In this study we performed QTL analysis using an F_2_ population derived from a cross with a new *R*-donor showing broad spectrum resistance to BB races in Korea. The QTL analysis confirmed that the *R*-gene was within the target region identified from the association mapping of the JMAGIC population, and is located in a region of high ORF density on the long arm of chromosome 11. A DNA marker specific to this newly identified *R*-gene was developed in this study, and could prove useful in the future development of BB resistant breeding lines using MAS. However, given that the location of the new BB *R*-gene is in close proximity to many candidate *R*-genes, further research is warranted to identify the specific gene and its function, to advance the understanding of molecular mechanisms of BB resistance in rice.

## Materials and methods

### Plant materials

A total of 120 *japonica* MAGIC (JMAGIC) [23] lines out of a total population of 381 lines, and the eight parents of the JMAGIC population (P1-P8) were used for association mapping based on the phenotypic measure of BB lesion length on inoculated leaves. The mapping population for QTL analysis was developed from a cross between P8 and Ilpum. A total of 451 F_2_ individuals were produced from the pure F_1_ plants of the parents and subsequently used for genotyping and phenotyping. The parental line, P8 (Colombia XXI; IRGC: 126955) is one of eight parents of JMAGIC population [23] showing resistance to Korean BB isolates and Ilpum is a high quality *japonica* cultivar (cv.) in Korea which is susceptible to BB and does not harbor any known BB resistance genes [19]. F_1_ plants from the same parents were used to develop a backcross population for fine mapping. Selected BC_2_F_1_ plants were identified by PCR using SSR markers, and 831 BC_2_F_2_ individuals were produced by self-pollination.

### Evaluation of BB resistance

A total of 17 virulent strains of *Xoo* from Korea were used to evaluate the spectrum of BB resistance of P8. Of these strains, the *Xoo* isolate K3a (HP01009) was used for phenotypic analysis of the mapping population consisting of the F_2_ individuals. To evaluate the BB resistance, the parents, F_1_ progenies and the F_2_ population were inoculated using leaf-clipping method [24] at maximum tillering stage of plants under green house and field conditions in Iksan and Jeonju, Korea. Leaf damage caused by the pathogen was evaluated 14 days after inoculation (DAI) according to the standard evaluation methods of the Rural Development Administration (RDA), Korea [25]. Evaluation of leaf damage level by the pathogen was conducted by measuring of the average lesion length of three leaves (Resistant: <3cm, moderately resistant: 3-5cm, susceptible: >5cm). Both resistant and moderately resistant phenotypes were classified as demonstrating resistance in this study.

### Association mapping

120 JMAGIC lines were selected and phenotyped by measuring lesion length of leaves inoculated with BB race K3a. The Infinium 6K Bead Chip was used for genotyping the tested lines. The SNP chips were composed of 384-SNP sets customized for *indica-japonica* SNP chip (ID: GS0011862-OPA), and the results were scanned using the Illumina BeadXpress Reader (Genotyping services Lab, IRRI). GWAS analysis was carried out based on a mixed linear model (MLM) using genome association and prediction integrated tool (GAPIT) in R [26]. PCA.total = 3 was applied as number of principal components (PCs) to use to control for population structure.

### Linkage analysis and QTL mapping

DNA samples of plant materials were prepared for genotyping of the mapping population following Murray and Thompson [27]with minor modifications. To construct a genetic linkage map, SNPs showing polymorphic patterns within the parents were identified from the Infinium 6K chip and these polymorphisms obtained from the parent survey were adopted for constructing linkage map using QTL IciMapping version 4.0 [28]. The segregation distortion of selected SNPs for linkage mapping was identified using the MAP functionality of the IciMapping software compared to the expected segregation ratio (1:2:1). Mapping distance for linkage map was calculated by recombination frequency with Kosambi mapping function and the functional option on the software, “By LOD” and “By Input”, were applied for grouping and ordering of anchored SNPs. The mean lesion length of three leaves was used to detect QTLs related to the resistance of individuals to BB. For QTL analysis the conventional Interval Mapping for additive QTL (IM-ADD) and inclusive composite interval mapping for additive QTL (ICIM-ADD) were adopted with SNP genotypic data and the phenotypic measure of lesion length of tested lines. The permutation LOD value was used as the threshold to declare the significance of the QTLs and the threshold from permutation was defined at *P≤0*.*05*.

### Development of markers for narrowing down the target region

Markers for detecting InDel polymorphisms were developed within the target region using the DNA polymorphism database [29]. The STS marker was developed based on the sequence from Rice Annotation Project Database (RAP-DB, http://rapdb.dna.affrc.go.jp/) and cleaved amplified polymorphic sequence (CAPS) was designed through detecting the specific restriction site according to sequencing data of STS-PCR products. A total of 110 PCR-based DNA markers (S1 Table) were tested to identify polymorphisms in parents of the mapping population. Eleven DNA markers located within the region were then selected from the RAP-DB.

### RNA isolation and quantitative real-time PCR

To prepare total RNA, leaf tissues were collected from mock-infected plant (0h) and in 1, 2, 4, and 24h after the inoculation, respectively. Total RNA was extracted from rice seedlings using the TRIzol reagent (Invitrogen, Carlsbad, CA, USA) according to the manufacturer’s protocols. The amfiRivert cDNA Synthesis Platinum Master Mix (GenDEPOT, Barker, TX, USA) was used for cDNA synthesis according to protocols provided by the manufacturer. Diluted cDAN was analyzed by using Stratagene MX3005P qPCR System (Agilent) and amfiSure qGreen Q-PCR Master Mix (GenDEPOT, Barker, TX, USA) for gene expression. To evaluate transcript levels, the rice *eEF1-α* gene was used as an internal control for qRT-PCR data normalization [30]. Each set of experiments was repeated three times, and the ddCT relative quantification method was used to evaluate the quantitative variation.

## Results

### Leaf reaction to different BB isolates

Eight parents and the 120 JMAGIC lines were screened for reaction to BB race K3a by measuring lesion length of leaves following inoculation. Only two of the parents, P6 and P8, demonstrated resistance to the isolate used for testing. The distribution of lesion length, showing the leaf reaction pattern to K3a for the lines is shown in S1 Fig. For further evaluation of the resistance profile of the *R*-gene in P8, the susceptible *japonica* cv. Ilpum and P8 were screened for resistance to a range of BB races. A total of 17 Korean BB isolates were used for the evaluation (Table 1).

**Table 1:** Leaf reaction of tested plants to 17 different BB isolates.

Lines	Reaction to different BB isolate (cm)																
	HB 1009	HB 1013	HB 1014	HB 1015	HB 2010	HB 2024	HB 2038	HB 3034	HB 3055	HB 4030	HB 4032	HB 4040	HB 4052	HB 4074	HB 4084	HB 4087	HB 5004
P8	1.5	2.0	0.5	2.0	0.5	2.5	3.5	4.5	3.5	3.0	3.5	4.0	4.5	4.0	4.0	4.5	4.0
Ilpum	12.0	10.1	7.0	8.0	10.0	12.5	5.5	12.3	10.5	12.5	10.3	10.0	7.5	12.3	11.3	6.7	6.0

The donor parent P8 was resistant or moderately resistant (lesion length < 5 cm) to all tested isolates, while Ilpum was susceptible to most of isolates (Table 1). In particular the donor demonstrated a strong resistance to four main BB races, K1 (HB01013), K2 (HB01014), K3 (HB01015) and K3a (HB01009), which are the most widespread, representing 95% of BB incidence in Korea

### Phenotyping for BB

**Fig 1:**
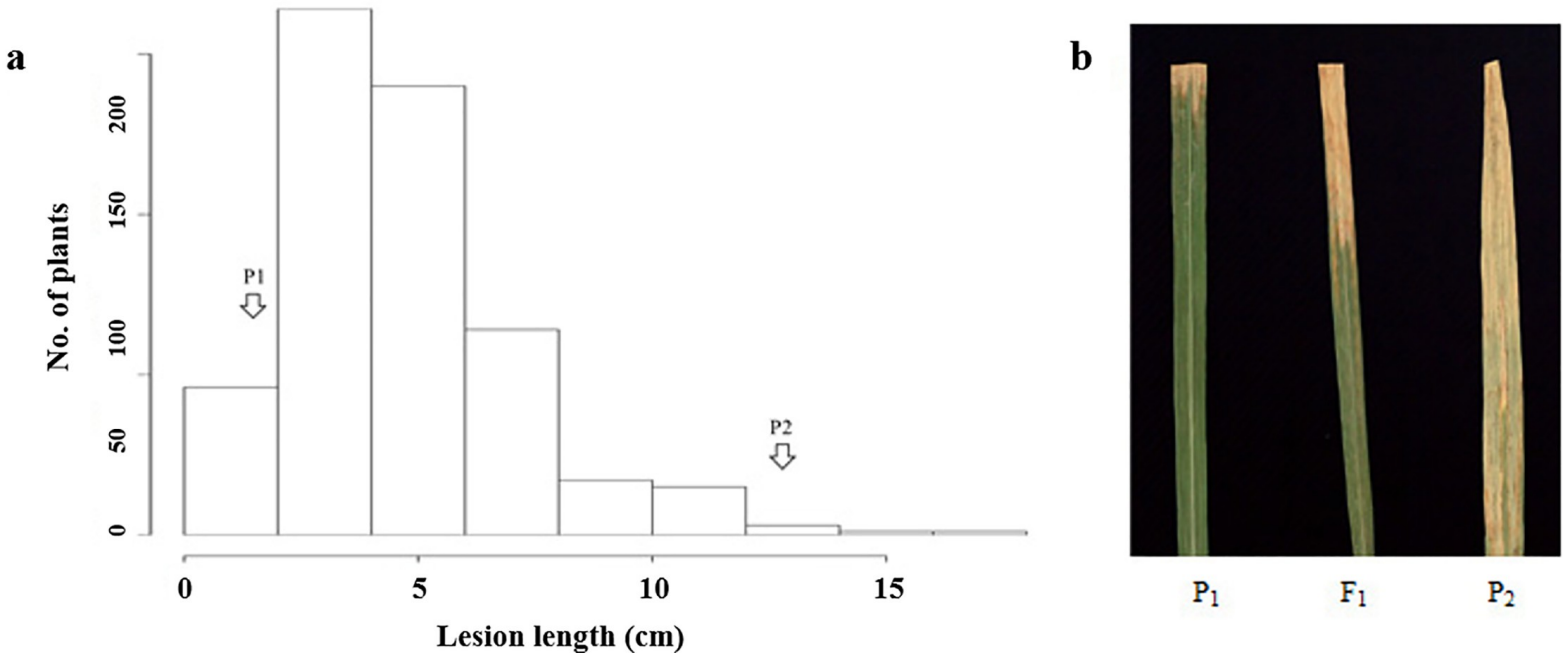
Disease reaction of a population of 451 F_2_ plants and parents to BB race K3a. a is distribution of lesion length of the F_2_ mapping population and parents at 14 DAI. b is resistant phenotype of parents and F1 plant. P1 is the resistant donor and P_2_ is susceptible donor.

The resistance of the P8/Ilpum F_2_ population, parents, and F_1_ plants to BB race K3a were evaluated and the phenotypic data used for QTL analysis (Fig 1).

The donor parent P8 was resistant to the K3a race with lesion lengths of less than 2.5 cm at 14 DAI, while Ilpum was susceptible with lesion lengths of 13 cm or greater at 14 DAI. F_1_ plants were confirmed using SSR markers showed moderate resistance to the K3a, with lesion lengths of 5.2 ± 0.8 cm. The 451 plants tested in the F_2_ population had lesion lengths ranging from 0 cm and 25 cm. The frequency graph of BB resistance showed a continuous distribution from 1 to 20 cm, and was slightly skewed to left (resistance) (Fig 1). The segregation ratio of the F_2_ population was 349 resistant and 112 susceptible, respectively. The observed allelic frequency fitted the expected phenotypic ratio of 3:1 (*X*^2^ = 4.15 and *P* = 0.12, *P*>0.05). For the BC_2_F_2_ population, the segregation ratio also followed the expected segregation ratio for a recessive gene of 3:1 (R593:S238), with *X*^*2*^ = 6.16 and *P* = 0.015 (*P*>0.01). These findings confirmed a single dominant resistance gene conferring *Xoo* resistance in P8.

### Detection of the target region and linkage mapping

**Fig 2:**
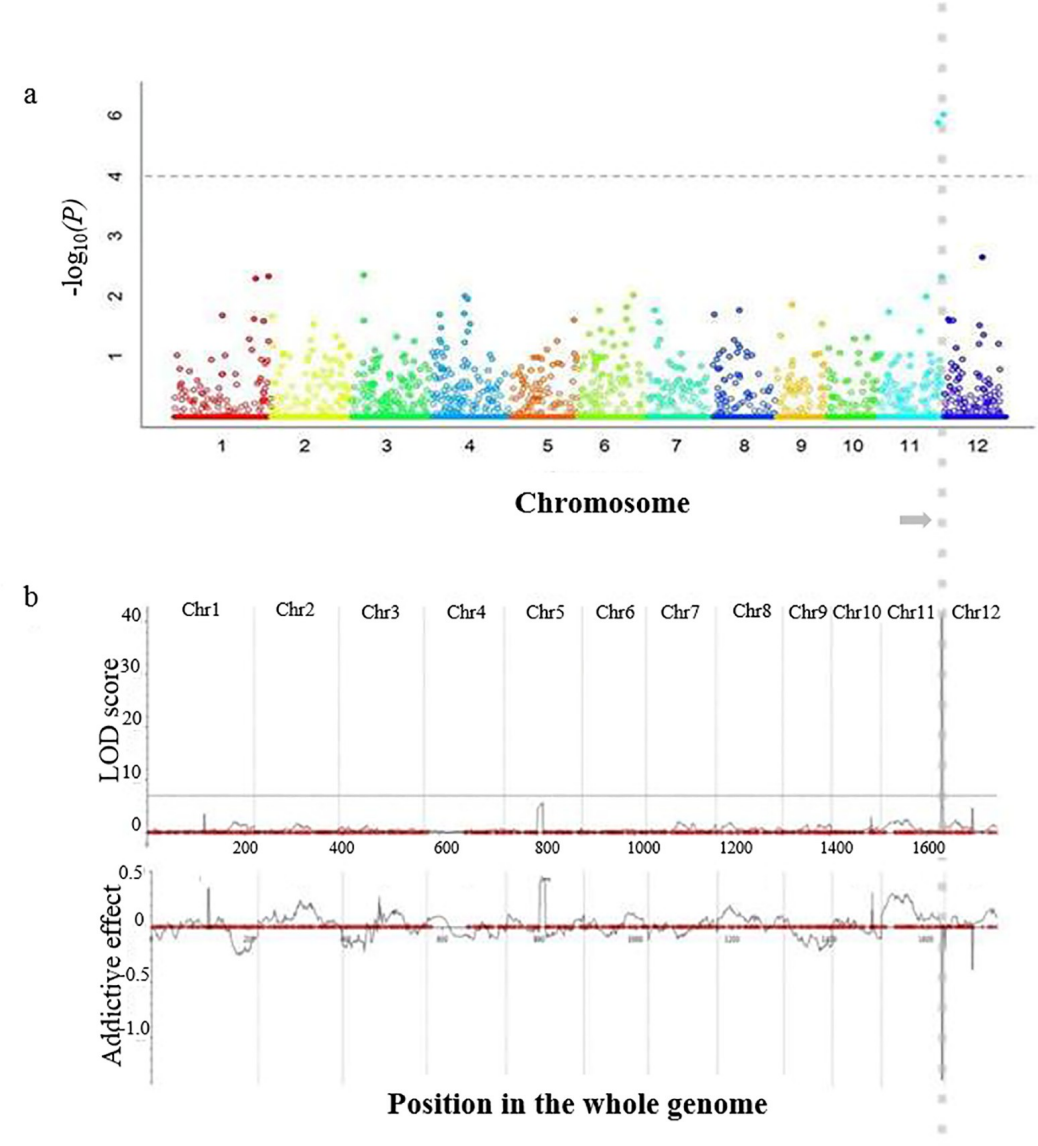
*R*-gene mapping by association and QTL analysis. a is Manhattan plot produced by GWAS analysis with genome association and prediction integrated tool (GAPIT). The x-axis indicates genomic position of each SNP and the y-axis is negative logarithm of *p*-value, obtained from GWAS model. Large peaks on chromosome 11 suggest that the surrounding genomic region has a strong association with the BB race, K3a (p < 0.05) and QTNs marked with grey vertical dotted line. b is LOD profile and additive mapping using inclusive composite interval mapping (ICM) on whole rice chromosome. The SNP location was positioned as red circle on the position in the whole genome. Positive additive effects were derived from susceptible parent Ilpum and negative values were related to the resistant parent. The horizontal grey line is the LOD threshold calculated by 1,000 permutations and the vertical dotted lines indicated by the grey arrow show the loci from both analysis is identical on the chromosome 11.

To detect quantitative trait nucleotides (QTNs) associated with BB resistance within the population of 120 JMAGIC lines, association mapping was conducted using results from the 6K SNP chip analysis. The detected QTNs were delimited within two SNPs, 1192907 and 11943779 (Fig 2A and S2 Table). To confirm the target region by GWAS subsequent QTL mapping with a bi-parental population was carried out using the 6K SNP chip for generating genotypic data. Of a total of 4,606 SNPs a total of 1,596 polymorphic markers were finally selected according to segregation distortion, stacked position of SNPs and their distribution, for constructing the genetic map (Table 2).

**Table 2:** Distribution of polymorphic SNPs across 12 rice chromosomes from parental survey.

Chromosome	Tested markers	Selected markers	Polymorphism rate (%)
Chr1	514	216	42.0
Chr2	457	174	38.1
Chr3	486	139	28.6
Chr4	421	87	20.7
Chr5	378	138	36.5
Chr6	376	127	33.8
Chr7	367	156	42.5
Chr8	345	127	36.8
Chr9	300	114	38.0
Chr10	276	113	40.9
Chr11	346	108	31.2
Chr12	340	97	28.5
Total	4,606	1,596	34.6

The range of polymorphism rate was from 20.7% to 42.5% with an average of 34.6% on whole chromosomes. The software IciMapping v4.0 with the Kosambi function was used to construct a high density genetic linkage map for this study, with a mean of 133 markers anchored on each chromosome (S2 Fig). The genotyping map data resulted in a total length of 1,684cM and an average of 1.1cM distance between SNP markers, and this was combined with the phenotypic data generated following inoculation with the K3a BB race, to identify the resistance gene to BB race K3a.

### QTL analysis

Genotypes of the F_2_ individuals were analyzed using 1,596 out of the 6K SNP chips and these data combined with the phenotypic data of the population were used for QTL analysis. From the analysis five QTLs were detected on chromosomes 1, 5, 10, 11, and 12, respectively, however the LOD scores of detected QTLs, except *qBB11* on chromosome 11, were less than the threshold LOD score. The threshold LOD scores of 6.9 for CIM and 7.0 for IM, respectively, were established using 10,000 permutations in IciMapping at significance levels of *P ≤* 0.05. The QTL, *qBB11*, on the long arm of chromosome 11 had an LOD value above the threshold computed by permutation analysis (Fig 2B). *qBB11* was consistently detected by IM and CIM in the software analysis and the percentage of phenotypic variation (*R*^*2*^) explained was 31.25% in ICIM and 31.35% in IM analysis, with LOD values of 43.44 and 36.06 harboring the two flanking SNP markers, 11943779 and 11963686 (Table 3).

**Table 3:** Putative QTLs associated with BB resistance gene detected by composite and interval mapping.

Analysis	QTLs	Chr.	Position(cM)	L marker	R maker	LOD	*R*^*2*^(%)	Add	Dom
ICIM	*qBB11*	11	126	11943779	11963686	43.44	31.25	-1.37	-0.1
IM	*qBB11*	11	126	11943779	11963686	36.06	31.35	-1.34	-0.52

ICIM: inclusive composite interval mapping, IM: interval mapping

*R*^*2*^: Percentage of phenotypic variation explained by the QTL

Add: additive effect

Dom: dominance effect

The detected QTL was also confirmed with nature of the QTL effect being a mixture of additive effects (-1.37 and -1.34) and dominant effects (-0.1, -0.52) in both ICIM and IM analyses respectively. Both the QTN and QTL were shown to be the same locus on chromosome 11 through GWAS and QTL mapping.

### Identification of target regions

**Fig 3:**
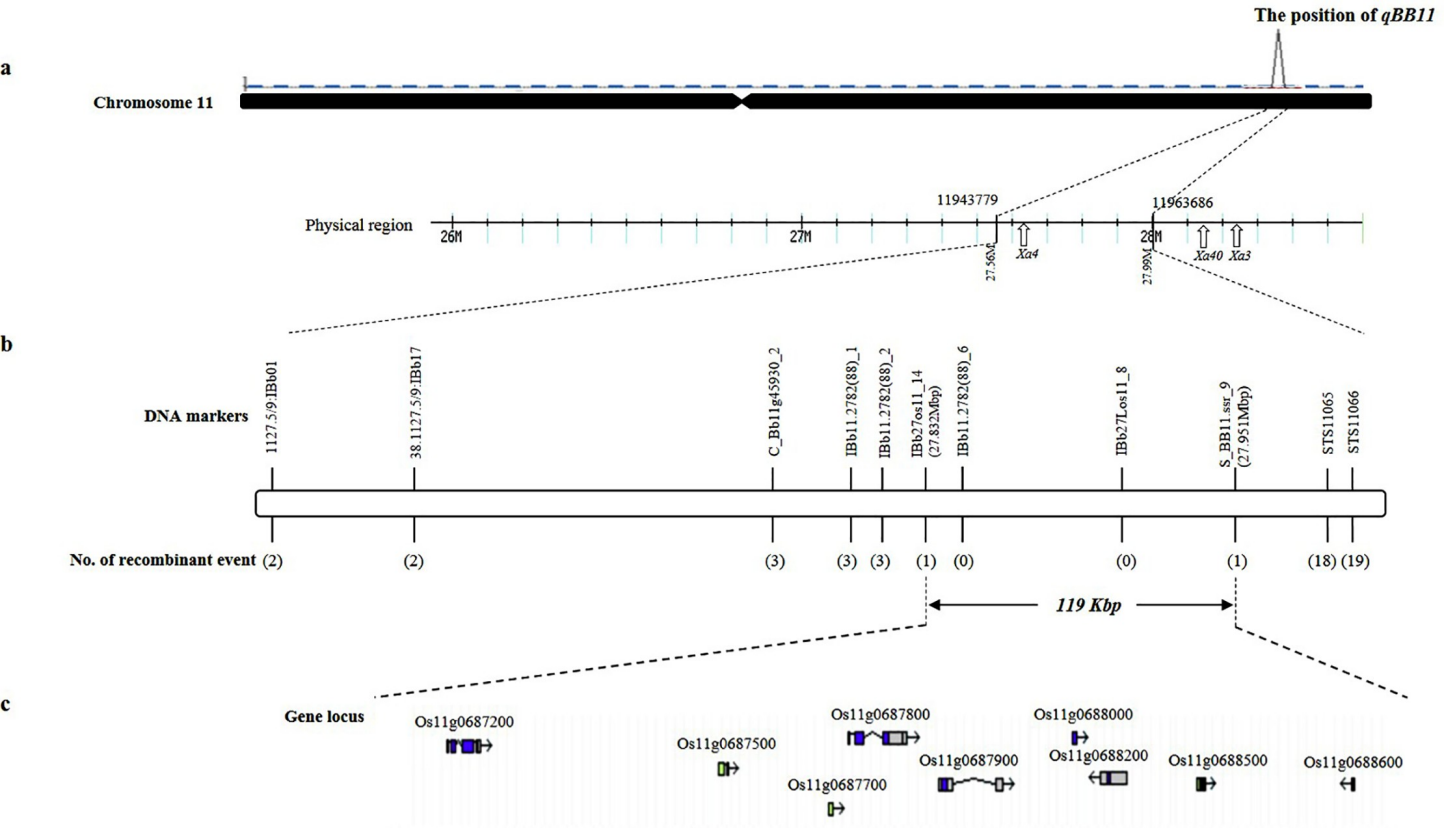
Dissection of *qBB11* regions. a is physical position of flanking markers and known BB *R*-genes on the chromosome 11 based on the Nipponbare genome. b is the position of the novel *R*-gene anchored by two DNA markers IBb27os11_14 and S_BB11.ssr_9. The number of recombinant events detected in the BC_2_F_2_ individuals were marked in brackets. c is gene locus listed on RAP-DB (IRGSP-1.0) based on the fine mapping. Nine candidates for the BB R-gene were included in approximately 119-Kbp.

The QTL was mapped on chromosome 11, and included a target region defined by flanking markers 11943779 and 11963686 which were located at 27.56Mbp and 27.99Mbp (Fig 3A). Additional markers were developed to dissect the interval containing *qBB11* (S1 Table). Of 110 markers (61 InDel, 35 STS, 12 SSR, and 2 Caps marker) newly designed for fine mapping, eleven markers showed polymorphism between the parents. To delimit the physical location of the target region BC_2_F_2_ individuals were used and twenty recombinants were selected based on the discordance by genotype and phenotype data (S3 Table). The selected DNA markers were anchored on the target region flanked by DNA markers 1127.5/9:IBb01 (27.56Mbp) and STS11066 (27.99Mbp), respectively (Fig 3B). According to the number of recombinant events, the region was further narrowed down and the flanking region for the *qBB11* delimited by two DNA markers IBb27os11_14 and S_BB11.ssr_9, defining a segment of approximately 119 Kbp (Fig 3B). Nine ORFs located in the target region were identified as candidate genes conferring resistance to BB (Fig 3C). Os11g0687500 and Os11g0687700 encoded expressed proteins, Os11g0688000 and Os11g0688200 encoded conserved hypothetical proteins and Os11g0687200 encoded a hypothetical protein. Os11g0687100, Os11g0687800, and Os11g0687900 encoded von Willebrand factor, NB-ARC domain containing protein and similar to MLA10 protein (S4 Table).

### Expression analysis of candidate genes in the target region

**Fig 4:**
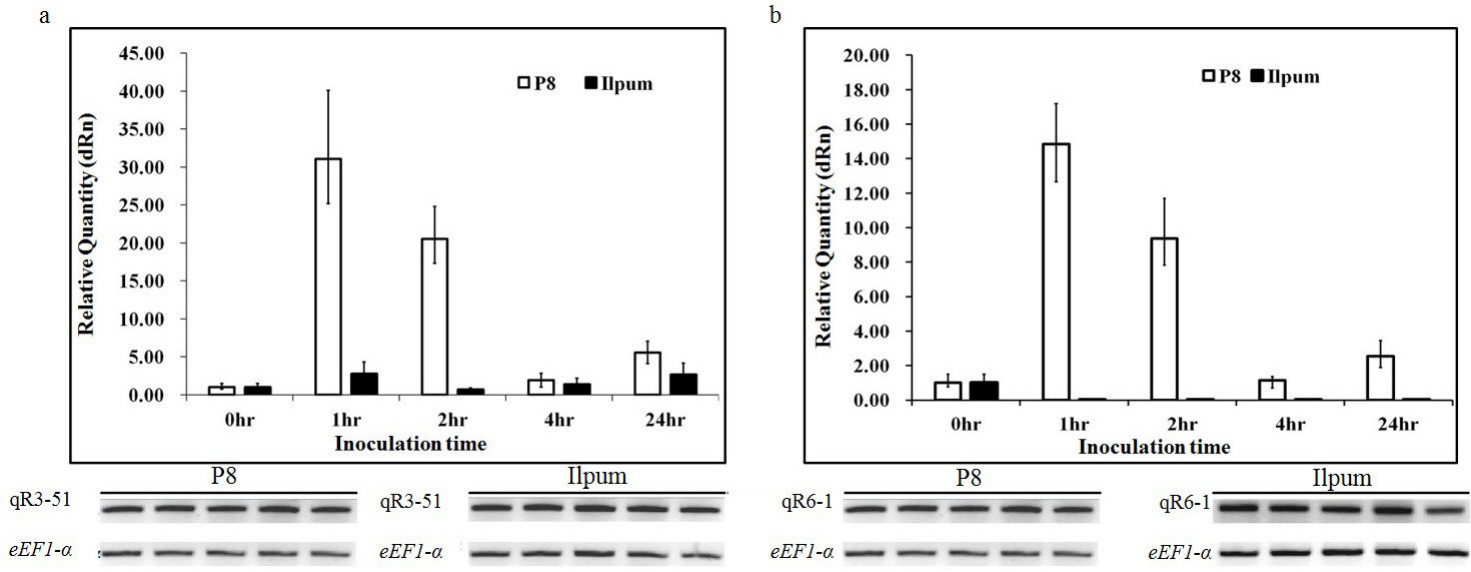
**Gene expression of Os11g0687700 (a) and Os11g0688000 (b) by qRT-PCR of P8 and Ilpum after BB inoculation.** mRNA expression levels of Os11g0688000 was analyzed at five time-points (0, 1, 2, 4, and 24h) after inoculation.

To analyze expression levels of the candidates an additional primer set was designed based on the genetic sequence using RAP-DB (S5 Table). Of the nine candidates, up-regulation of expression levels in P8 was observed in two ORFs Os11g0687700 and Os11g0688000 through qRT-PCR analysis (Fig 4 and S6 Table). In particular, the expression level of Os11g0687700 in P8 at 1h after inoculation revealed significantly up-regulated expression at a level of about 32 times of the concentration at 0h (Fig 4A), while remaining seven ORFs exhibited no significant difference of expression levels in the parents (data not shown).

## Discussion

The bacterial leaf blight (BB) caused by *Xoo* is one of the most widespread and devastating diseases in most rice cultivation areas. To date about 42 BB resistance genes have been identified and these are commonly used in breeding for BB resistance [20]. Given the relatively common breakdown of BB resistance caused by occurrence of a new BB pathotype or change of *Xoo* population it is necessary to find new resistance genes and combine these with known *R*-genes in order to develop durable and sustainable resistant lines.

In this study, we performed QTL analysis using an F_2_ population developed from a cross between P8, the BB *R*-donor, and Ilpum, a susceptible *japonica* cultivar, to detect a novel BB *R*-gene. The bi-parental mapping combination was developed based on the previous study using JMAGIC lines, and fine mapping and qRT-PCR were subsequently carried out to further identify the novel BB *R*-gene.

The JMAGIC population is a multi-parental population developed by IRRI to be used as a resource to map QTLs for multiple traits, and to use the highly recombined lines in breeding programs [23]. To identify resistant lines to Korea BB races we used the JMAGIC population and screened the eight parents along with 120 JMAGIC lines to evaluate the degree of resistance. From the analysis of the JMAGIC lines, we found a locus related to BB resistance on one end of chromosome 11 (Fig 2A). The *R*-gene(s) was harbored within the flanking SNPs, 11943779 (27.56 Mbp) and 11992907 (28.73 Mbp), on chromosome 11 (S2 Table). In general, association mapping can complement linkage mapping and facilitate fine QTL mapping [31]. However, the population used in the association mapping was comprised of lines from the JMAGIC population selected primarily according to days to heading, from a total of 381 JMAGIC lines, and was a relatively small population size for a linkage or association study. Thus, to avoid false positives from the associated locus we decided to combine association mapping with linkage analysis in order to be able to confirm the loci related to BB resistance.

To validate the locus for BB resistance an F_2_ population derived from a cross between P8 and Ilpum was developed and was used for constructing a linkage map and for QTL analysis in this study (S2 Fig and Table 3). In the genetic analysis using 451 F_2_ and 831 BC_2_F_2_ individuals the *R*-gene showed segregation ratios consistent with a single gene with dominant gene action.

The *R-*donor (P8) used in this study showed resistance to all tested races in the bioassay conducted using 17 different BB isolates, including the isolate HB 1009 (K3a), in Korea (Table 1), thereby demonstrating a broad-spectrum resistance to all tested races. The parents and F_1_ plants demonstrated resistance to K3a at 14 DAI with an average lesion length of 1.0, 13, and 5.2cm, respectively (Fig 1). Given the segregation ratios of the F_2_ population (*R*:*S* = 3:1) and the reaction of the F_1,_ this suggested that the resistance could be controlled by a major gene with incomplete dominance. In resistance studies this type of inheritance is frequently reported in rice [19,32–34].

The target region detected by association mapping using GWAS was confirmed by QTL analysis of a bi-parental mapping population in this study (Fig 2). Based on the linkage analysis one major QTL associated with BB resistance was identified on chromosome 11 and the QTL *qBB11* had a high LOD value of 43.44, explaining 31.25% by CIM of the phenotypic variation (Table 3).

To further narrow down *qBB11* delimited by flanking SNPs, fine mapping was conducted using 831 BC_2_F_2_ individuals, and the novel *R*-gene *Xa43(t)* was delimited to an approximately 119-Kbp segment flanked by DNA markers IBb27os11_14 and S_BB11.ssr_9. The locus narrowed down by fine mapping was located nearby a region known to harbor three BB R-genes, *Xa3*, *Xa4* and *Xa40*. However the positions covering *R*-genes were not physically identical each other [19,35,36]. In addition, *Xa3* has a susceptible reaction to K3a (data not shown) and *Xa43* also has a different haplotype to *Xa40* and *Xa4* based on testing with a primer specific to *Xa43* (S3 Fig). In additional race-specific bioassays, *R*-reaction between *Xa43* and *Xa4* was different when exposed to the HB3011 and HB2010 BB races (S7 Table). According to the fine mapping we identified nine ORFs involved in the target region and qRT-PCR was performed to confirm the expression level of these nine candidates using additional primer sets developed based on the exons of candidates. Expression levels of two ORFs Os11g0687700 and Os11g0688000 in P8 were significantly higher than that of Ilpum (Fig 4).

From the results we surmise that the *R*-gene *Xa43 (t)* might be a novel gene involved in conferring BB resistance in rice and suggest the likelihood of both may be considered candidate genes for *Xa43(t)*. The two ORFs exhibited vastly increased expression levels within the target region identified by fine mapping, encoding uncharacterized proteins.

## Conclusions

In this study we confirmed the location of a novel BB resistance gene on chromosome 11 using QTL analysis and fine mapping. The QTL detected, *qBB11*, was renamed *Xa43(t)* and the physical location delimited to an approximately 119-Kbp segment by PCR based DNA markers IBb27os11_14 and S_BB11.ssr_9 on chromosome 11. Through qRT-PCR analysis two ORFs (Os11g0687700, and Os11g 0688000) demonstrated significant up-regulation of expression levels, noted in the resistant variety P8 in comparison with the susceptible variety Ilpum. Considering the propensity of *R*-genes to break down, and the need to counter race differentiation in disease resistance breeding, we hope this result will contribute to increasing the spectrum of *R*-genes available, the identification of new and durable *R*-genes, and facilitate development of combinations of *R*-genes using MAS. In particular, we suggest the PCR-based DNA marker may find practical use for MAS breeding programs to improve BB resistance in rice, with the combination of multiple sources of resistance contributing to more durable resistance, leading to greater stability of rice production in areas afflicted by bacterial blight.

## Supporting information

S1 Fig Lesion length of 120 JMAGIC lines and their 8 parents to BB race K3a. The blue bars indicate the distribution of lesion lengths among the tested lines at 14 DAI. P1 (CSR30), P2 (Cypress), P3 (IAC166), P4 (Jinbu), P5 (WAB 56–126), P6 (IR73571-3B-11-3-K3), P7 (Inia Tacuari), P8 (Columbia XXI).
(PDF)

S2 Fig Genetic linkage map of the 12 chromosomes based on 1,596 SNP markers segregating in the P8/Ilpum 451 F_2_ population. (PDF)

S3 Fig Comparison of amplicon size derived from five different lines. P6 and P8 are *R*-donor parents of the eight JMAGIC parents, resistant to BB races. Junam, IRBB4 and 11325 have *Xa3*, *Xa4*, and *Xa40(t)*, respectively. M is 100bp size marker.
(PDF)

S1 Table DNA markers tested for fine mapping. (XLSX)

S2 Table Result of GWAS on reaction to BB isolate HB1009 using GAPIT. (XLSX)

S3 Table Comparison of genotypic and phenotypic data of 20 recombinants within the target region. Green cells show concordance and the violet cells reflect discordance between phenotypic and genotypic data.
(XLSX)

S4 Table List of putative genes in the garget region. Gene locus has transcript evidence or protein homologs and predicted locus consists of predicted genes without any transcript evidence.
(XLSX)

S5 Table Primer sets designed for qRT-PCR. (XLSX)

S6 Table Analysis of Ct values of housekeeping gene and target gene expression at 0hr, 1hr, 2hr, 4hr and 24hr after inoculation of bacterial blight for P8 and Ilpum. (XLSX)

S7 Table Leaf reaction of four lines to 10 BB isolates at 14 days after inoculation. P8, IRBB4, and 11325 have *Xa43*, *Xa3*, *Xa4*, and *Xa40(t)*, respectively, and Ilpum was used as a susceptible check. Average lesion length was obtained after measuring three inoculated leaves.
(XLSX)
