# Supplementary material for: A novel resistance gene for bacterial blight in rice, Xa43(t) identified by GWAS, confirmed by QTL mapping using a bi-parental population
Source: PLoS One. 2019 Feb 12;14(2):e0211775. doi: 10.1371/journal.pone.0211775 (PMC6372157; doi:10.1371/journal.pone.0211775)
Supplement: S1 Fig — The blue bars indicate the distribution of lesion lengths among the tested lines at 14 DAI. P1 (CSR30), P2 (Cypress), P3 (IAC166), P4 (Jinbu), P5 (WAB 56–126), P6 (IR73571-3B-11-3-K3), P7 (Inia Tacuari), P8 (Columbia XXI). (PDF) [file pone.0211775.s001.pdf]

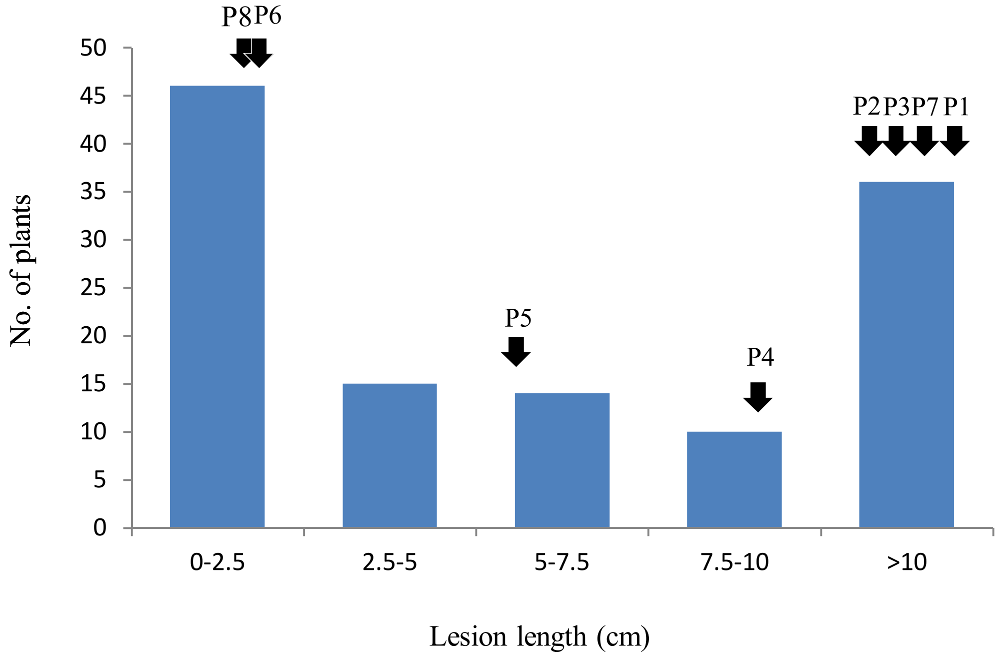

S1 Fig. Lesion length of 120 JMAGIC lines and their 8 parents to BB race K3a. The blue bars indicate the distribution of lesion lengths among the tested the lines at 14 DAI. P1(CSR30), P2(Cypress), P3 (IAC166), P4 (Jinbu), P5 (WAB 56-126), P6 (IR73571-3B-11-3-K3), P7 (Inia Tacuari), P8 (Columbia XXI).
