# Supplementary material for: A novel resistance gene for bacterial blight in rice, Xa43(t) identified by GWAS, confirmed by QTL mapping using a bi-parental population
Source: PLoS One. 2019 Feb 12;14(2):e0211775. doi: 10.1371/journal.pone.0211775 (PMC6372157; doi:10.1371/journal.pone.0211775)
Supplement: S2 Fig — (PDF) [file pone.0211775.s002.pdf]

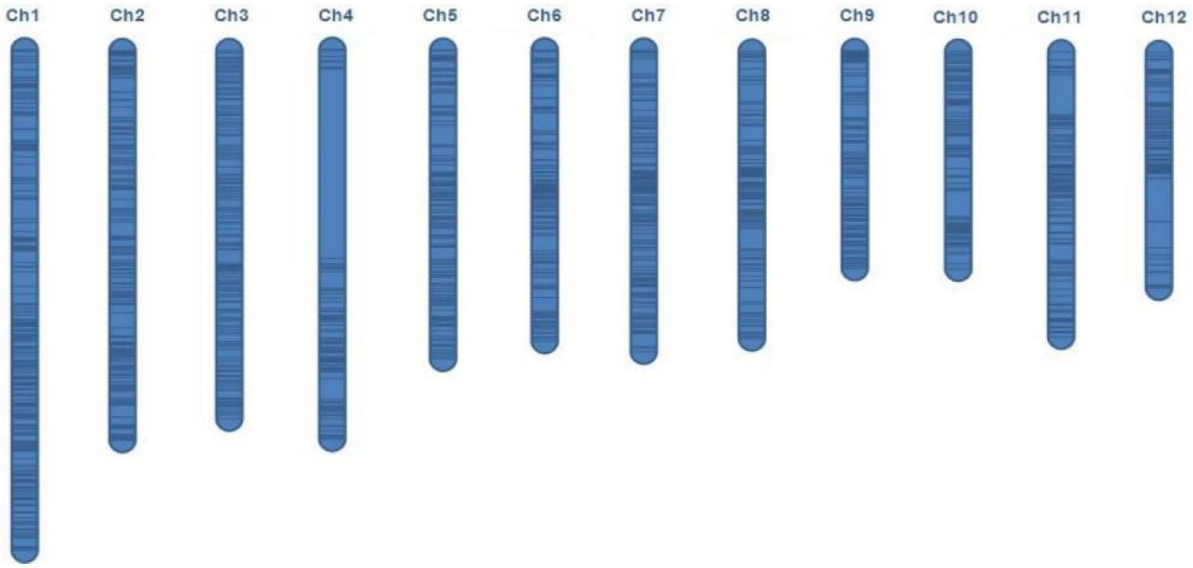

S2 Fig. Genetic linkage map of the 12 chromosome based on 1,596 SNP markers segregating in in the P8/Ilpum 451 F_2 population.
