# Supplementary material for: A novel resistance gene for bacterial blight in rice, Xa43(t) identified by GWAS, confirmed by QTL mapping using a bi-parental population
Source: PLoS One. 2019 Feb 12;14(2):e0211775. doi: 10.1371/journal.pone.0211775 (PMC6372157; doi:10.1371/journal.pone.0211775)
Supplement: S3 Fig — P6 and P8 are R-donor parents of the eight JMAGIC parents, resistant to BB races. Junam, IRBB4 and 11325 have Xa3, Xa4, and Xa40(t), respectively. M is 100bp size marker. (PDF) [file pone.0211775.s003.pdf]

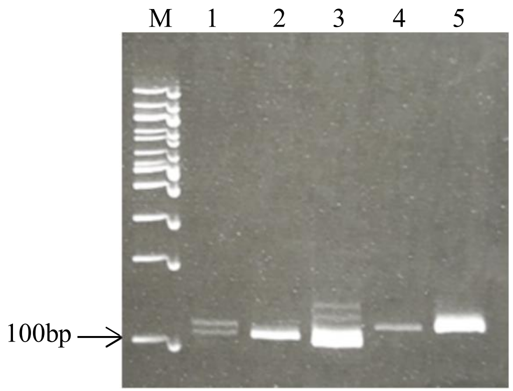

1: P6, 2.P8, 3:IRBB4, 4:Junam, 5:11325

S3 Fig. Comparison of amplicon size derived from five different lines using DNA marker IBb27os11_14. P6 and P8 are *R*-donor parents out of eight in JMAGIC lines , which showed resistant to BB races. Junam, IRBB4 and 11325 have *Xa3*, *Xa4*, and *Xa40(t)*, respectively. M is 100bp size marker.
